# Nanotribological Properties of Graphite Intercalation Compounds: AFM Studies

**DOI:** 10.1155/2017/9438573

**Published:** 2017-11-02

**Authors:** Zhiwei Chen, Dan Guo, Lina Si, Guoxin Xie

**Affiliations:** ^1^State Key Laboratory of Tribology, Tsinghua University, Beijing 100084, China; ^2^School of Mechanical Engineering, Beijing Institute of Technology, Beijing 100081, China

## Abstract

Tetraalkylammonium salts have larger ions than metal ions, which can greatly change the interlayer space and energy, and then potentially tune the properties of graphite. In this work, various graphite intercalation compounds (GICs) have been synthesized by intercalating tetraoctylammonium bromide (TOAB) ions into graphite through electrochemical interactions under different reduction potentials. Different degrees of expansion between graphite layers as well as their corresponding structures and topographies have been characterized by different analytical techniques. The nanoscale friction and wear properties of these GICs have been investigated by AFM-based nanofrictional and scratch tests. The results show that electrochemical intercalation using tetraalkylammonium salts with different interaction potentials can tune the friction and wear properties of graphite. Under relatively large applied loads of AFM tips, friction increase and wear can be easier to occur with the increase of the intercalation potential. It is inferred that the increases of both the interlayer space of graphite and the number of ions on the surface give rise to puckered effect and formation of rougher surfaces. This work gives us deep insight into the friction and wear properties of GICs as composite lubrication materials, which would be of great help for material design and preparation.

## 1. Introduction

Graphite is a good choice for intercalation host and has many unique applications, such as highly conductive materials and battery electrodes [1–3]. It shears easily along the direction parallel to the planes due to weak van der Waals interactions between layers, resulting in excellent lubricant properties [4–6]. Thus, it is possible to modify the interlayer force of graphite for the changes in friction and wear properties of graphite. Graphite intercalation compounds (GICs), formed by inserting ionic species into the lattice of the graphite structure, are typically layered materials consisting of intercalate guest and graphite layers [7, 8]. The distance between the layers in GICs can be changed, and then the interlayer force of graphite can be modified. There have been dense studies about the syntheses, compositions, and properties of GICs with various intercalate species [9–11]. The syntheses methods of GICs include electrochemical and chemical methods, and the GICs can be produced by oxidation or reduction reactions [12–15].

Many researches in the past few years have shown that there are different ways to intercalate different ions into graphite. The intercalate ions are mainly alkali metal ions and organic ammonium ions (R_4_N^+^). A lot of chemical methods have been applied for the syntheses of R_4_N^+^ and alkali metal ions GICs via cationic displacement reactions [16, 17]. Recently, the structure, synthesis, electrical behavior, and physical properties of heavy alkali metal-arsenic alloy-based GICs have been investigated using traditional chemical methods. It has been found that the intercalated atomic sheets are highly dependent on the synthesis conditions [18, 19]. Compared with traditional chemical methods, electrochemical procedures for charging graphite electrodes are relatively simple but less common. Three different methods of graphite intercalation have been compared in recent researches, and it comes out that compared to the Hummers method and the gas phase methods, the electrochemical method provides a simple, effective, and potentially cheap way for intercalation [20]. Graphite cathodes are inert in aqueous solutions because the water electrolysis potential limits the working potential of graphite cathodes [21]. Aprotic medium is comparatively stable during the cathodic reduction, so it would not be affected at large negative potentials [22]. Tetraalkylammonium cations (TAAs) are usually chosen as electrolytes in nonaqueous electrochemical experiments due to their excellent solubility in organic solvents. The intercalation of TAAs in graphite has been reported previously, and the electrochemical reduction method has been adopted in polar aprotic organic solvents such as 1,2 dimethoxyethane, dimethylsulfoxide (DMSO), N,N-dimethylformamide (DMF), and propylene carbonate [23–25]. Recently, a series of TAA/GICs have been prepared in DMSO-based TAA electrolytes using cathodic reduction methods, and the compositions and structures of the GICs have been characterized [26, 27].

So far, there have been many studies on micro, nanoscale friction and wear properties of the graphene. It has been reported that graphene can reduce friction compared with the surrounding substrate, and its friction can be effectively modulated by functionalization or changing the layer number [28]. It also has been found that ultralow friction can be achieved by immersing graphite in water [29]. Recently, it has been found that ultrasound treatment of expanded graphite (EG) can increase wear resistance due to strengthening of the running film and enhanced adhesion of the transfer films [30]. The AFM scratching test has been applied to explore the wear properties of graphene, and emphasis has been put on the plastic deformation of graphene by plowing parallel trenches [31]. The mechanical properties of graphene-covered Pt(111) surface also have been studied by computer simulations and experiments. The results show that graphene elastically deforms at low loads, while plastic deformation of the Pt below the graphene layer occurs at intermediate loads, and eventually, graphene ruptures at high loads [32].

Although many studies about the wear of graphite and the synthesis of GICs have been conducted, the understanding of the friction and wear of GICs is still superficial. In our previous work, it has been found that the friction forces of the synthesized TAA/GICs are almost one-third of those of the pristine graphite at different scales [33]. In order to deepen our understanding on the tribological properties of GICs, the friction and wear properties of TAA/GICs prepared with different intercalate potentials have been studied at nanoscale under relatively large applied pressures on the basis of AFM experiments. Then more insights can be gained about the consistencies and discrepancies of tribological properties of GICs at different scales.

## 2. Experimental Methods

### 2.1. Materials

Graphite powder (average particle diameter 150 *μ*m, 99.95%, metals basis) was purchased from Macklin Biochemical Co., Ltd. (Shanghai, China). Copper mesh (500 mesh) was purchased from Huawei hardware store (Beijing, China). Ag/AgCl electrode and platinum mesh electrode were purchased from Ida Co., Ltd. (Tianjin, China). Tetraoctylammonium bromide (TOAB, purity > 98%) was purchased from J&K Scientific Co., Ltd. (Beijing, China). Dimethylsulfoxide (DMSO, AR grade, 99.9%, dehydrated over a 4 Å molecular sieve prior to use) and anhydrous methanol (AR grade, 99.8%) were purchased from Modern Oriental Technology Development Co., Ltd. (Beijing, China).

### 2.2. Syntheses of GICs

**Figure 1:**
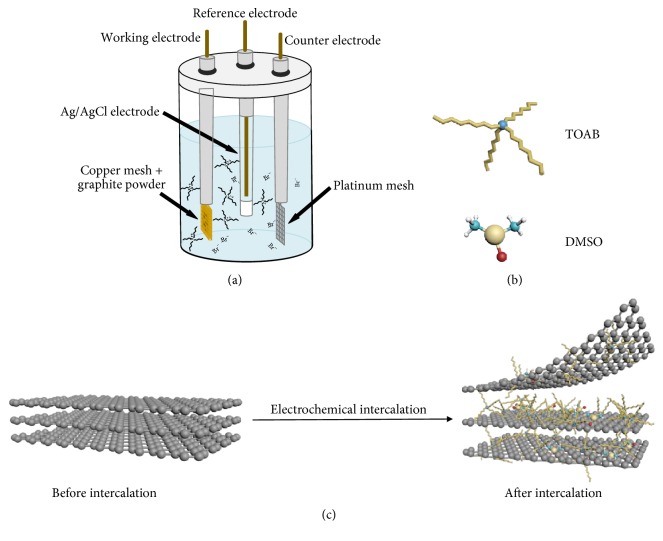
(a) The schematic diagram of the experimental cell for electrochemical intercalation of graphite powder; (b) the molecular structures of the intercalation molecule and the solvent molecule used in this work; (c) the schematic diagram of the change of the molecular structure of graphite during electrochemical intercalation.

Inorganic/organic GICs were synthesized with facile electrochemical intercalation process (see Figure 1). In order to make the working electrode, graphite powder was wrapped up in copper mesh, preventing the power from dispersing into solution. The Ag/AgCl electrode was used as the reference electrode and the platinum mesh electrode was the counter electrode. All the potential values were given versus the saturated Ag/AgCl reference electrode. TOAB was the intercalation substance, and DMSO was the solvent. Constant reduction potential and cyclic voltammetry was applied by CHI660C electrochemical workstation for the electrochemical intercalation reaction. The effects of electrolysis potential, electrolysis time, and electrolyte concentration of TOAB were explored. The results show that the intercalation substance could not be intercalated into the graphite in the cases of small electrolysis potentials or low electrolyte concentrations, and the larger electrolysis potential, the longer electrolysis time, and the higher electrolyte concentration of TOAB would give rise to sufficient intercalation reaction [33]. Hence, different GICs would be produced by choosing different electrolysis potentials, electrolysis times, and electrolyte concentrations. After explorations of the parameters, it was determined that the electrolysis time was 2 hours and electrolyte concentration was 0.1 M in DMSO. After electrochemical intercalation, the product was taken out from the copper mesh into a 200 mL clean centrifugal tube.

### 2.3. Preparation of Few-Layer GIC Nanoflake

**Figure 2:**
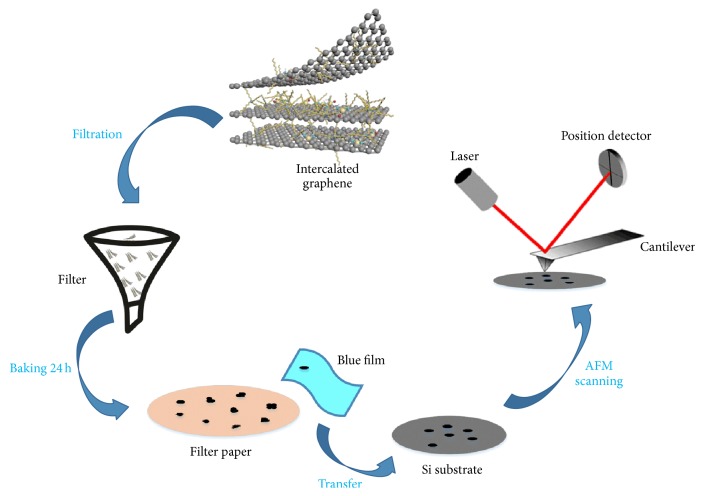
The schematic diagram of the postprocessing, mechanical exfoliation and transfer process of the product for AFM-based experiments.

In order to remove soluble byproducts and other impurities, the products were filtered three times with anhydrous methanol washing, and then they were baked in the vacuum drying oven for 24 hours. Few-layer GIC flakes were prepared by mechanical exfoliation where blue film adhesive tape was used to peel off the powder on the filter paper, and then the GIC flakes were transferred onto the Si substrate at room temperature (see Figure 2), which would expose the fresh surface to air for AFM height images and AFM-based frictional experiments.

### 2.4. Structural Characterizations

The X-ray diffraction (XRD) used Rigaku Miniflex II diffractometer with Ni-filtered Cu K*α* radiation with 5°/min scan speed and 2 *θ* ranged from 3° to 60°. The Raman spectra were obtained using Raman spectroscopy (LabRam HR 800) with Ar+ laser (514.5 nm). The relative humidity and temperature were 25°C and 10% in the experiment. The laser power was ~0.4 mW, which could reduce the local heating effect of laser beam on GIC flakes [34]. The spot size of the laser beam was ~1 *μ*m which could ensure the laser beam exerting right on small GIC flakes. The data of Raman spectra were analyzed by Origin software.

### 2.5. Atomic Force Microscopy

The atomic force microscopy (AFM) images were obtained by a Cypher (Asylum Research, US) instrument. Contact mode was utilized to obtain the height and friction images of the GIC flakes. The relative humidity and temperature were 25°C and 10%. One DCP20 probe (NT-MDT) with a normal force constant of 90.26 N/m was adopted in the AFM-based wear experiments of graphene. It was installed only once during every group of AFM wear experiments (with loads from 500 nN to 8000 nN) and removed after the completion of all the experiments to ensure the results were comparable. The tip was hardly to be worn out because of the presence of diamond coating. The improved wedge method proposed by Varenberg et al. [35] was used to calibrate the AFM frictional signal, and the obtained torsional constant was 1863 nN/V for the DCP20 probe used in this work.

## 3. Results and Discussion

### 3.1. Results of Structural Characterizations

**Figure 3:**
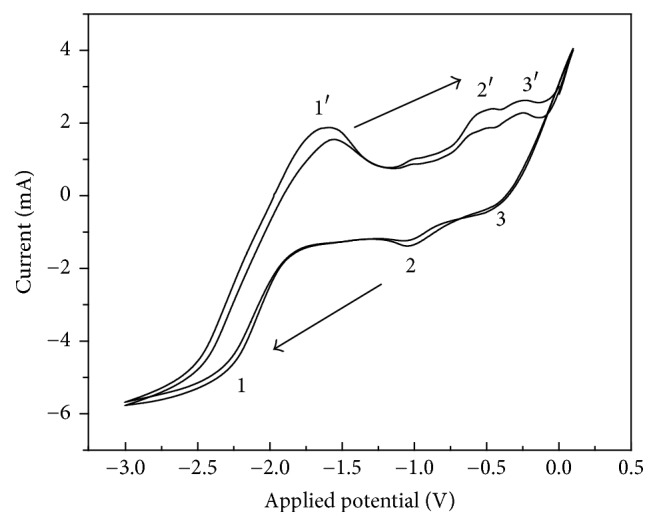
Cyclic voltammetry diagram (1st cycle and 2nd cycle) of graphite intercalation with potential from −3 V to 0.1 V, scan rate 5 mV/s, and start potential 0 V in 0.1 M DMSO solution.

The synthesized GICs were baked for 24 h in the vacuum drying oven in order to remove the residual solvent. Afterwards, they were used for subsequent characterizations, AFM friction, and wear experiments. Cyclic voltammetry experiments have been carried out. The CV curves of two cycles of graphite in 0.1 M DMSO solution with TOAB as electrolyte are shown in Figure 3. The scan rate is 5 mV/s and the start potential is 0 V. Anodic peak 2′ is related to the change of Cu into Cu^1+^, and anodic peak 3′ related to the change of Cu^1+^ into Cu^2+^. Cathodic peak 3 is related to the change of Cu^2+^ into Cu^1+^ and cathodic peak 2 related to Cu^1+^ changing into Cu. Peaks 1 and 1′ are related to the intercalation and deintercalation reaction of (C_8_H_17_)_4_N^+^. It could be observed that the onset of TOAB intercalation is about −1.6 V.

**Figure 4:**
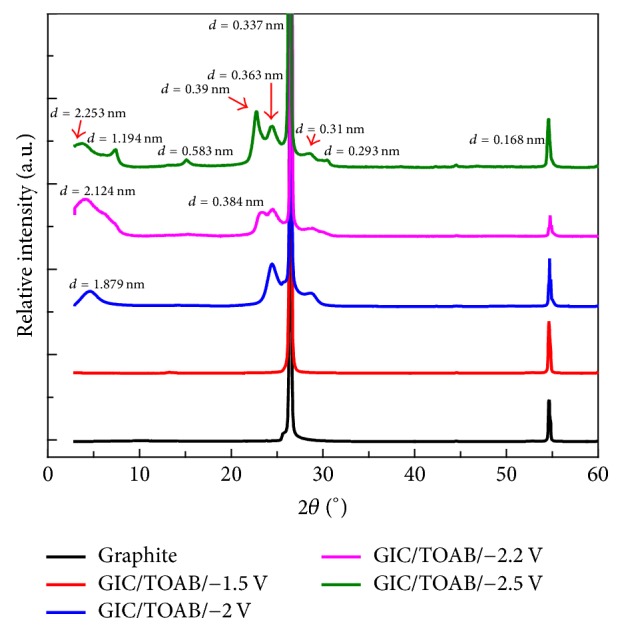
XRD patterns of pristine graphite powder, GIC/TOAB at the intercalation potentials of −1.5 V, −2 V, −2.2 V, and −2.5 V. All samples were dried in a vacuum oven for 24 h.

Figure 4 shows the XRD patterns of the synthesized GIC flakes. It shows that there is no new peak or peak shift for the GIC/TOAB flakes synthesized at a potential of −1.5 V during the electrochemical intercalation process, suggesting there was no obvious difference between GIC/TOAB at −1.5 V and graphite, and the electrochemical intercalation was not successful. New diffraction peaks appear for the GIC/TOAB flake synthesized at a potential of −2 V. The 2*θ* angles of the new diffraction peaks are 4.7°, 24.6°, and 28.8°, corresponding to the* d-*spacings of 1.879, 0.363, and 0.31 nm. The results indicate that (C_8_H_17_)^4^N^+^ cations have been successfully intercalated into the layers of the graphite powders. Besides, two new peaks have larger* d-*spacings than those of the pristine graphite power, suggesting that the expansion of the graphite interlayer is not uniform. It can be attributed to the number of intercalated (C_8_H_17_)^4^N^+^ cations that are random and the cations that are flexible. It is noteworthy that there is a new peak with a smaller* d*-spacing than the pristine graphite powder. It indicates that the ammonium cations were successfully intercalated into the inner layers of the graphite powder, giving rise to the increase of part of the interlayer *d*-spacings. Meanwhile, there were some interlayers with decreased* d*-spacings due to the squeezing of the expanded layers. As the potential was increased to −2.2 V, the diffraction peaks become clearer. The 2*θ* angles of new diffraction peaks are 4.2°, 23.1°, 24.5°, and 28.9°, corresponding to the* d-*spacings of 2.124, 0.384, 0.363, and 0.31 nm. It suggests that more (C_8_H_17_)^4^N^+^ cations were intercalated into the graphite powder for the GIC/TOAB synthesized at −2.2 V than those of the GIC/TOAB synthesized at −2 V. As compared with the GIC/TOAB synthesized at −2 V, there is a new peak at 23.1° for the GIC/TOAB synthesized at −2.2 V, indicating that the expansion was more nonuniform. The new 23.1° peak may be formed partly due to the red-shift of 24.5° peak. The most left peak moved from 4.7° to 4.2°, and the* d-*spacing increased, suggesting the expansion of the GIC/TOAB synthesized at −2.2 V is more sufficient than that of the GIC/TOAB synthesized at −2 V. When the potential was increased to −2.5 V, the diffraction peaks become more complex. The 2*θ* angles of the new diffraction peaks are 3.9°, 7.4°, 15.2°, 22.8°, 24.4°, 28.6°, and 30.5°. As compared with the GIC/TOAB at −2.2 V, the three new peaks at 7.4°, 15.2°, and 30.5° imply the nonuniform expansion in the GIC/TOAB at −2.5 V. The most left peak moves from 4.2° to 3.9°, and the 23.1° peak moves to 22.8°, suggesting that the expansions are more obvious than the GICs/TOAB at −2 V and −2.2 V.

The size of (C_8_H_17_)N^+^ is about 1.4 nm [27], and *d*_1_ for GIC intercalated by (C_8_H_17_)N^+^ cations is 1.148 nm [26]. The generated GICs could be classified in different stages I, II, III,…, *n*, where the meaning of stage *n* is that one intercalant layer follows after *n* graphene layers. The relationship of the gallery height (*d*_i_), repeat distance along* c*-axis (*d*_n_), and stage number (*n*) is given by [26]

(1) $$\begin{array}{c} d_{n}=d_{i}+0.335\left(\;n-1\;\right). \end{array}$$

It can be calculated [36] that 2.124* d*-spacing XRD peak corresponds to stage 4 (001) GICs, 0.384* d*-spacing peak corresponds to stage 1 (003) GICs, 0.363* d*-spacing peak corresponds to stage 3 (005) GICs, 0.583* d*-spacing peak corresponds to stage 1 (002), and 1.194* d*-spacing peak corresponds to stage 1 (001). Some stage 1 peaks appear when the potential is −2.5 V, indicating that the −2.5 V GICs are more fully intercalated than −2 V GICs.

Because the onset of TOAB intercalation is about at −1.6 V according to the cyclic voltammetry diagram, the GICs fabricated at −2 V for 2 h are sufficient. Hence, the mass fraction of TOAB would not increase obviously even though the potential is increased to −2.5 V. According to the XRD patterns, most of the expanded layers have* d*-spacing of 0.363 nm at −2 V, and more than half of these layers expand to* d*-spacing of 0.39 nm when the potential is −2.5 V, suggesting that the mass ratio is bigger than 11%.

Overall, with the increase of the potential applied during the electrochemical intercalation of graphite powder, the intercalation was more sufficient, and the nonuniform expansions of interlayers are more obvious because of the randomness of the number and the molecular flexibility of intercalated cations. In addition, the amount of the peaks suggesting the increase of* d-*spacings is more than that of the peaks suggesting the decrease of* d-*spacings for all of the GICs, implying that the expansion of interlayers is more obvious than the compression of interlayers.

**Figure 5:**
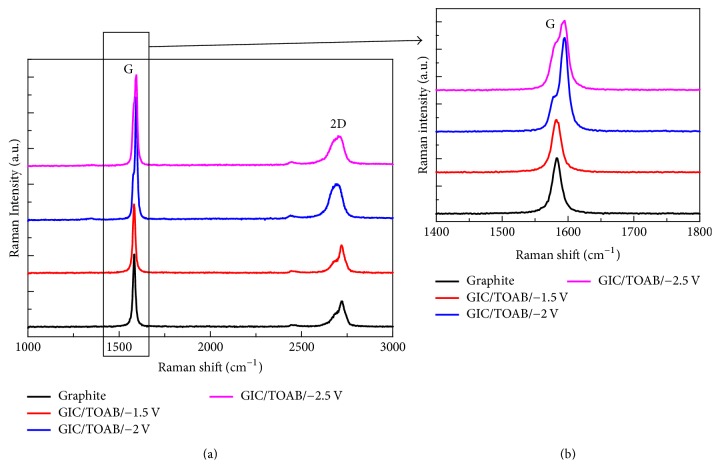
(a) Raman spectra of pristine graphite powder, GIC/TOAB with the intercalation potentials of −1.5 V, −2 V, and −2.5 V; (b) the details of the G peak shown in (a).

In order to get more structural information of the GIC powder, Raman spectra analyses were used, and the results are shown in Figure 5. The Raman spectrum of the pristine graphite powder shows a wide peak at ~1580 cm^−1^, and it is the G peak exhibiting the doubly degenerate zone center E_2 _g vibration mode [37]. The Raman spectrum of GIC/TOAB synthesized at −1.5 V is similar to that of graphite powder. It means that there was almost no difference between the GIC/TOAB at −1.5 V and the pristine graphite powder. The G peaks of GICs/TOAB at −2 V and −2.5 V partly move to the right, which could be considered as new peaks with a combination of the ~1580 cm^−1^ peak and the ~1600 cm^−1^ peak. The shifted G peak is associated with the energy shift, which was caused by the electron transfer from the graphene layers to the intercalated cations [33, 36, 38, 39]. Furthermore, the 2D peak of GIC/TOAB at −1.5 V is similar to pristine graphite powder, which indicates the intercalation was insufficient for GIC at −1.5 V. Red shifts of the 2D peaks of the GICs/TOAB at −2 V and −2.5 Vas compared to those of pristine graphite powder can be observed. It means that there are intercalant layers between graphene layers [40], also indicating that the intercalation was successful for the GICs at −2 V and −2.5 V.

### 3.2. AFM Topography and Friction Comparisons

**Figure 6:**
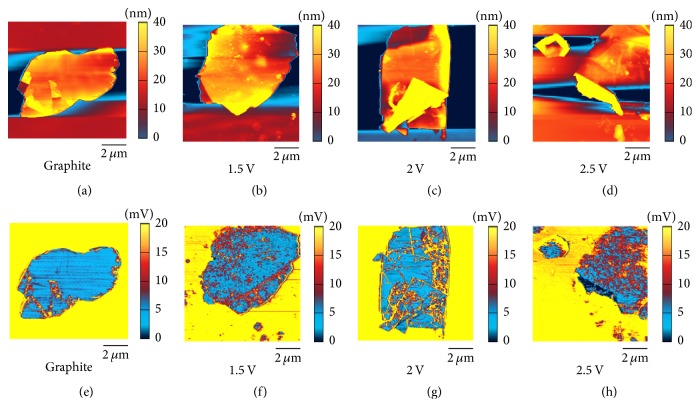
AFM height images of pristine graphite flake (a), GIC/TOAB flake at −1.5 V (b), GIC/TOAB flake at −2 V (c), and GIC/TOAB flake at −2.5 V (d). AFM friction images of pristine graphite flake (e), GIC/TOAB flake at −1.5 V (f), GIC/TOAB flake at −2 V (g), and GIC/TOAB flake at −2.5 V (h).

The AFM height and friction images were observed, and the results are shown in Figure 6. It could be observed in Figure 6(a) that the surface of pristine graphite flake is uniform and flat, and Figure 6(e) shows that the friction of the graphite surface is also uniform and is much smaller as compared with that of the nearby silicon substrate.  Figure 6(b) is the height image of the GIC flake at −1.5 V, and the surface is still flat with some small dots (intercalation substances) present on the surface. The corresponding friction image in Figure 6(f) shows that the intercalation substances have larger friction than that of pristine graphite, and the friction distribution is nonuniform due to the presence of intercalation substances.  Figure 6(c) shows that the edge of the GIC flake at −2 V expanded; it is because ammonium cations were intercalated into the graphite from the edge, but there was no sufficiently large driving force for these cations intercalating into the center part. Hence, the expansion of the edge part is more obvious than that of the center part. It could be observed that some upper layers have been turned over due to mechanical exfoliation during the preparation of the flake. The interlayer is not uniform which is caused by ammonium cations adhering to the interlayer.  Figure 6(g) shows that the friction distribution is not uniform, similar to the case shown in Figure 6(f), and the friction of the expanded edge is more nonuniform than that of the flat part, besides the friction is also nonuniform for the interlayers in the upper layers which were turned over.  Figure 6(d) exhibits that the edges expanded much larger than those of the GIC flakes at −2 V, which was caused by the larger driving force applied to the ammonium cations due to the larger potential.  Figure 6(h) shows that the friction distribution is not uniform, and the edges have large friction. It is due to the fact that a large quantity of intercalation cations was exposed to the edge due to excess intercalation, and the structures of the edges become uneven, giving rise to the increase of the roughness. However, the obviously expanded surface has the smaller friction than that of the flat surface.

**Figure 7:**
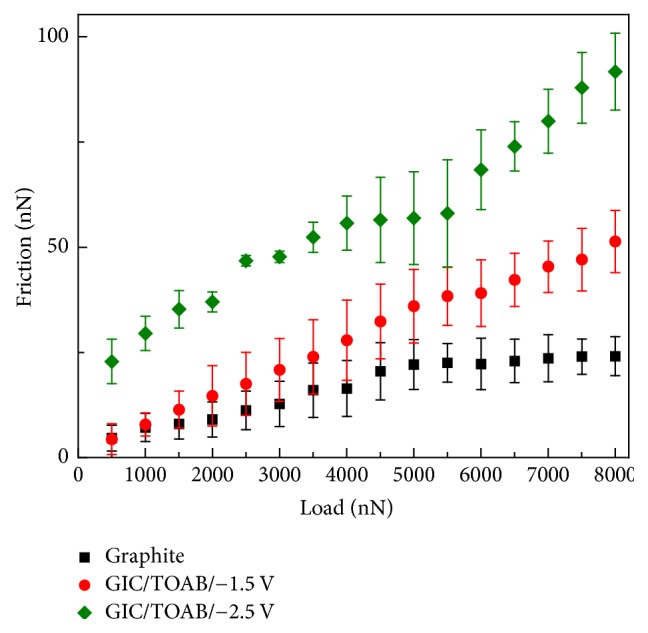
Plots of the friction of pristine graphite powder, GIC/TOAB at −1.5 V and GIC/TOAB at −2.5 V with load.

Previous experiments have demonstrated that the GIC flakes have been successfully produced. There are questions about what friction and wear properties these GIC flakes have. Hence, AFM friction experiments were carried out in this research. The friction data of graphite and different GIC flakes with loads from 500 to 8000 nN was shown in Figure 7. The friction was increased to some degree as compared with that of graphite and it also increases with load.

The nanoscale friction measured by an AFM tip at high loads is composed of the shear force at the tip-surface interface and the out-of-plane deformation [41]. When the load is large enough, there will be puckered effect along the sliding direction [42, 43] and the interlayer shear between the underlying layers will exist. Large interlayer spacing would increase the puckered effect, giving rise to the increase of interlayer shear force and out-of-plane deformation.

**Figure 8:**
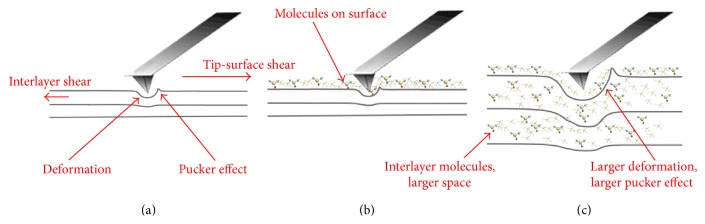
Schematic of the tip scratch process with high load on pristine graphite flake (a), −1.5 V GIC flake (b), and −2 V and −2.5 V GIC flake (c).

The friction of pristine graphite is lowest under the high load in Figure 7, and it is because the surface of pristine is smooth and the interlayer space is small, which makes pristine graphite has small roughness and interlayer shear (schematically shown in Figure 8(a)). The friction of the GIC/TOAB synthesized at −1.5 V is slightly larger than that of graphite. It is because its structure is similar to that of graphite due to insufficient intercalation, and there were only some residual ammonium ions and DMSO molecules on the surface, giving rise to the increase of the roughness, which leads to slight increase of friction (schematically shown in Figure 8(b)). The GIC/TOAB synthesized at −2.5 V has the highest friction. Massive ammonium ions have been intercalated into the interlayer of graphite when the potential is larger than −1.6 V, making the interlayer spacing large. It would increase the puckered effect, leading to large interlayer shear force and out-of-plane deformation. The interlayer intercalant molecules increase the interlayer roughness, which also increase the interlayer friction (schematically shown in Figure 8(c)). All these factors lead to the highest friction of −2.5 V GIC flake. If the tetraalkylammonium salts have shorted chains, the interlayer space would not be as large as that of long chain tetraalkylammonium salts because of smaller intercalation molecules. In this instance, the pucker effect decreases, and the friction would be smaller than that of long chain salts, but it would still be larger than pristine graphite [33].

### 3.3. Results of AFM Nanoscale Wear Experiments

**Figure 9:**
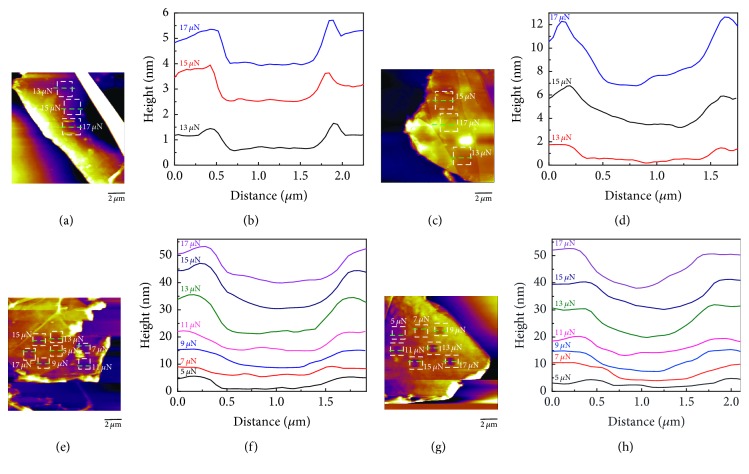
AFM height images of the surface scratches on the pristine graphite flake (a) and corresponding wear scars (b), GIC/TOAB flake at −1.5 V (c) and corresponding wear scars (d), GIC/TOAB flake at −2 V (e) and corresponding wear scars (f), and GIC/TOAB flake at −2.5 V (g) and corresponding wear scars (h). The thicknesses of these flakes were ~30 nm.

The nanoscale wear experiments on the basis of the AFM scratching method were also carried out. Each experiment lasted for 50 cycles in each square domain, and it means the probe slid 50 times at any given point in the wear domain. The scanning frequency for all the wear experiments was 10 Hz, and it took about 21 min in each square domain.  Figure 9 shows the morphologies of the different GIC flakes after AFM scratch experiments, and the thicknesses of the tested specimens were ~30 nm.  Figure 9(a) shows the topography of the pristine graphite which has some square domains after AFM scratching. There are square domains present when the normal loads were 13 *μ*N and 15 *μ*N, but it is hard to recognize because the depths of these domains are very low. The square domain is unclear even though the normal load was increased to 17 *μ*N. Hence it can be inferred that the graphite flake is hard to be worn out, because the interlayer structure of the graphite is compact, resulting in the increases of the hardness and strength of the graphite.  Figure 9(c) shows the topography of the GIC flake synthesized at −1.5 V after AFM scratching. The square domain is still unclear even though the normal load was applied to 17 *μ*N, indicating that the wear property of the GIC flake at −1.5 V is similar to that of graphite.  Figure 9(e) is the topography of the GIC flake at −2 V after AFM scratching. It can be seen that the depth of the square domain increases with the increase of the normal load, and the square domains become clearer at large normal loads. As compared with the square domains of graphite and the GIC flake at −1.5 V, the square domain of the GIC flake at −2 V is much more obvious under the identical normal load.  Figure 9(g) shows the topography of the GIC flake at −2.5 V. The square domain on Figure 9(g) is clear, and the depths of these square domains increase with the increase of the normal load. The wear property of the GIC flake at −2.5 V is similar to that of the GIC flake at −2 V except that the square domains on the GIC flake at −2.5 V are slightly clearer. These results indicate that the antiwear property deteriorates increasingly with the increase of the intercalation potential.

**Figure 10:**
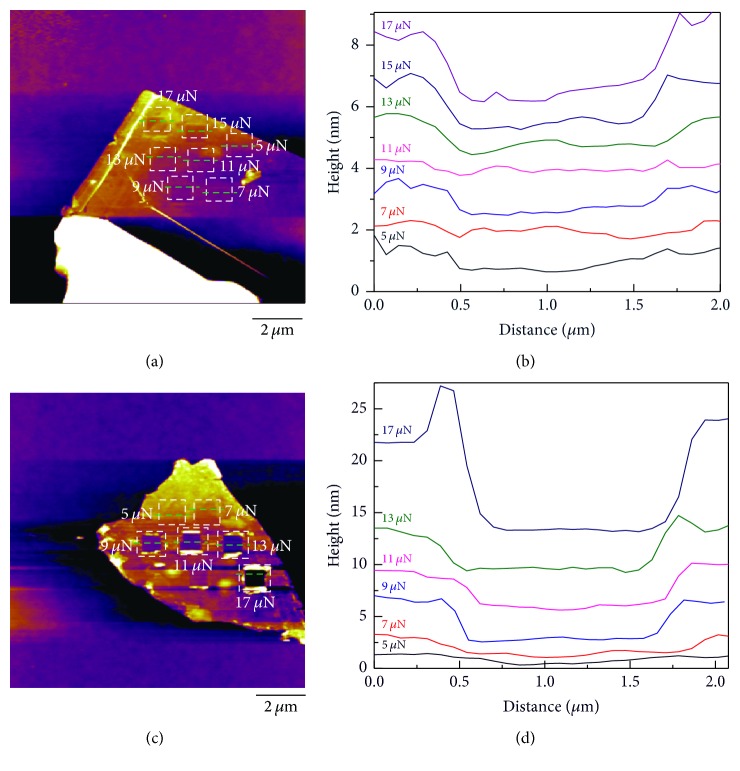
AFM height images of the scratches on the pristine graphite flake (a) and corresponding wear scars (b) and the GIC/TOAB flake at −1.5 V (c) and corresponding wear scars (d). The thicknesses of these flakes were ~10 nm.

It is hard to observe square domains in thick flakes because the ratio of the scratch depth to total flake height is relatively small, which is hard to get a good contrast. If the flake is thin, the ratio of the scratch depth to total flake height would increase dramatically, which is easier to get good contrast. Thinner flakes are more easily puckered in the out-of-plane direction due to tip-graphene adhesion, which could increase the frictional resistance, and thus the wear domains would be clearer. In order to get clear square domains on the surfaces of graphite and the GIC flake at −1.5 V, experiment was carried out on the thinner flakes (~10 nm), and the results are shown in Figure 10. It can be seen that the square domains become relatively clear on the surfaces of these thin flakes.  Figure 10(a) shows that the graphite flake with the thickness of ~10 nm has clear square domains than that with the thickness of ~30 nm. For the former, it is only plastically deformed when the load is less than 15 *μ*N. It could be observed in Figure 10(c) that the square domain is unclear for the GIC at −1.5 V and the flake did not fracture when the normal loads were 5 *μ*N and 7 *μ*N, indicating that the GIC flake at −1.5 V was only plastically deformed when the normal load was less than 9 *μ*N. The depth of the square domain increases, and more flakes were torn and peeled off with the increase of the normal load. As compared with the graphite flake, the GIC flake at −1.5 V was easier to be torn.

**Figure 11:**
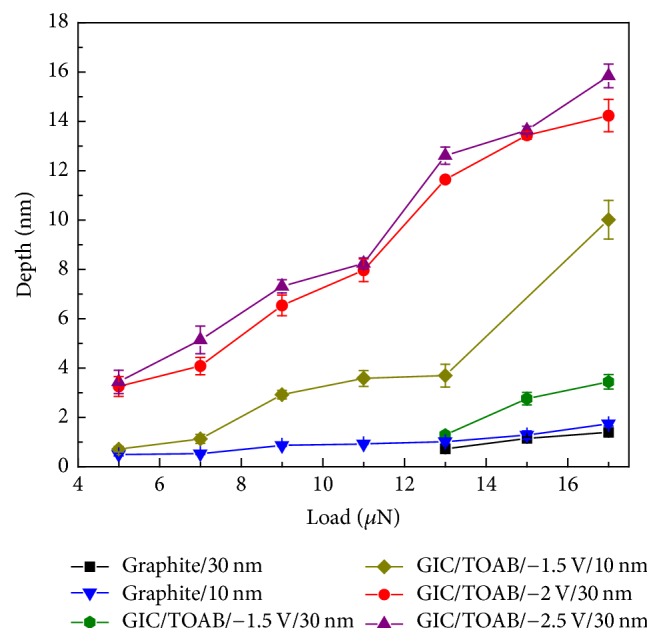
Plots of the wear depths against the applied load of the pristine graphite flakes, GIC/TOAB flakes at −1.5 V, −2 V, and −2.5 V with different thicknesses.

Variations of the wear depth as a function of the applied normal load are summarized in Figure 11. It could be observed that the GIC flakes with the thickness of ~30 nm at −2 V and −2.5 V have larger wear depths than that of the GIC flakes at −1.5 V and the graphite flake at all the investigated loads. The wear depths of the GIC flake at −2 V are similar to those of the GIC flake at −2.5 V. It indicates that successful electrochemical intercalation could make the structure of graphite loose and thus easier to get thinner flakes than the pristine graphite. And then scratch is easier on the GIC flake surface than on the graphite surface. The square domains of ~10 nm graphite flakes and thin GIC flakes at −1.5 V are slightly deeper than those of ~30 nm samples. It is because that the friction of graphene sheets increases with decreasing the thickness of samples. Thinner flakes are more easily puckered in the out-of-plane direction due to the tip-graphene adhesion, which could increase the frictional resistance. Thus, the wear resistance of thin flakes is different from thick flakes [44].

## 4. Conclusion

Electrochemical method has been used to intercalate tetraoctylammonium bromide ions into pristine graphite powder at different potentials of −1.5 V, −2 V, and −2.5 V. The sample surfaces become more uneven with the increase of intercalation potential, and obvious expansions of the edges can be observed after intercalations. On the basis of AFM-based nanofrictional tests, it has been found that the friction of these GICs increases with the intercalation potential under high loads. The wear properties of GICs have been measured by AFM scratch experiments, and the results show that the wear of graphite becomes easier with the increase of the potential. These results can be explained by the increase of both the interlayer space of graphite and the number of the ions on the surface after intercalation, leading to the increase of puckered effect. This work gives us deep insights into the friction and wear properties of GICs as composite lubrication materials and lubricant additives.
